# Prosthetist screening for comorbidity during routine care visits: a randomised controlled clinical trial evaluating benefits, acceptability and feasibility

**DOI:** 10.1136/bmjopen-2025-108623

**Published:** 2026-02-27

**Authors:** Jaclyn M Sions, Samantha J Stauffer, John R Horne, Ryan T Pohlig

**Affiliations:** 1 Department of Physical Therapy, University of Delaware, Newark, Delaware, USA; 2 Biomechanics and Movement Science Graduate Program, University of Delaware, Newark, Delaware, USA; 3 Independence Prosthetics-Orthotics, Inc, Newark, Delaware, USA; 4 Biostatistics Core, University of Delaware, Newark, Delaware, USA; 5 Epidemiology Program, University of Delaware, Newark, Delaware, USA

**Keywords:** Clinical Trial, Depression & mood disorders, Back pain, Diabetic neuropathy

## Abstract

**Objectives:**

Comorbidity among adults with lower-limb amputation exceeds that in the general population and contributes to poor outcomes. The primary objectives of this clinical trial were to explore benefits, acceptability and feasibility of comorbidity screening by prosthetists during routine follow-up visits. Our primary hypothesis was that when compared with standard-of-care (SOC) follow-up visits, standard-of-care + comorbidity screening (SOC+) would result in greater patient satisfaction and reduced prosthetic care minutes.

**Design:**

Randomised controlled clinical trial with mixed-methods approach.

**Settings:**

Prosthetic clinical practices.

**Participants:**

70 adults with unilateral lower-limb amputation.

**Interventions:**

Participants were randomly assigned to receive SOC or SOC+, which included assessment for depressive symptoms, suicidal ideation, moderate-to-high risk low back pain, lack of pedal pulses suggestive of peripheral arterial disease and lack of protective sensation suggestive of peripheral neuropathy. Screening results were reviewed with participants and faxed to primary care with telephone follow-ups when indicated. Prosthetists participated in a focus group. Healthcare utilisation over the subsequent 3 months was tracked.

**Primary and secondary outcome measures:**

Patient satisfaction surveys and care utilisation.

**Results:**

Few adverse events and protocol deviations occurred; intervention fidelity was >95%. There were no significant between-group differences in overall patient satisfaction, prosthetic care utilisation over the subsequent 3 months, nor initial prosthetic care visit length (p>0.050). Item-by-item analysis found participants rated prosthetist responsiveness to concerns higher with SOC+ (p=0.046, r=0.251). Prosthetists identified benefits from screening. Screening prompted positive healthcare seeking behaviours in this vulnerable population.

**Conclusions:**

Comorbidity screening by prosthetists appears feasible and acceptable.

**Trial registration number:**

NCT05410548.

**STRENGTHS AND LIMITATIONS OF THIS STUDY**

- Participants were randomly allocated to standard-of-care (SOC) or SOC plus screening.
- SOC procedures were developed and agreed on by prosthetists at the clinical practice.
- Intervention fidelity was assessed by the principal investigator; feedback was provided.
- But only one prosthetic practice with four prosthetists was included in the clinical trial.
- Further, the sample was limited to adults with unilateral lower-limb amputations.

## Introduction

 Of the more than 160 000 people who undergo lower-limb amputation (LLA) annually in the USA,^1^ up to 50% will not survive 5 years.^2^ This is, in part, due to high rates of comorbidities, such as peripheral arterial disease (PAD), which affects >50% of adults with LLA and increases the risk of cardiovascular disease.^3 4^ Additionally, more than a year after amputation, depression, suicidal ideation and low back pain (LBP) rates exceed those of the general population, occurring in up to 30%,^5^ 15%^6^ and 50%^7 8^ of adults, respectively. To improve outcomes following LLA, comorbidities need to be diagnosed and effectively managed.

In the general population, adults are not routinely screened for comorbidities, such as PAD and peripheral neuropathy (PN), leading to underdiagnosis and undertreatment.^9^ The onus for comorbidity screening falls largely on primary care providers. In the USA, patients spend, on average, 18 min with their primary care provider^10^; studies show reduced comorbidity screening when visits are <20 min.^11^

Thus, comorbidity screening following LLA may need to be a shared responsibility among the patient’s interdisciplinary healthcare team. For up to 87% of patients post-LLA, prosthetists are part of the care team.^12^ Certified prosthetist-orthotist scope of practice includes assessment of mental status, circulation, protective sensation and pain, as well as determination of need for referral to other healthcare professionals. LLA clinical practice guidelines recommend patients follow-up with their healthcare team at least annually,^13^ which is often initiated by patients when there is a prosthetic-specific need. Therefore, prosthetic follow-up visits may be an opportunity for routine comorbidity screening.

Unsurprisingly, evidence suggests that if a medical condition does not improve, patient satisfaction is lower.^14^ Lower patient satisfaction is associated with increased healthcare utilisation, which is explained by patient need-related factors, such as mental health status, comorbidities and pain.^15^ Patient satisfaction with prosthetist–orthotist care is generally high,^16^ but one area for improvement may be care coordination with other providers.^17^ Hence, the primary objectives of this randomised controlled clinical trial were to explore potential benefits, acceptability and feasibility of comorbidity screening by prosthetists during routine follow-up visits. Our primary hypothesis was that when compared with a standard-of-care (SOC) prosthetic visit, standard-of-care + comorbidity screening (SOC+) would result in (1) greater patient satisfaction at 3 months due to enhanced care coordination and (2) reduced prosthetic care minutes given concurrent medical needs were identified and addressed. Mitigating prosthetic visits for issues that may be best addressed by other medical providers reduces unnecessary business expenses as prosthetic practices receive bundled payments for the entire prosthetic process, that is, practices are not billing by visit like other medical providers.

## Methods

### Study overview

**Figure 1:**
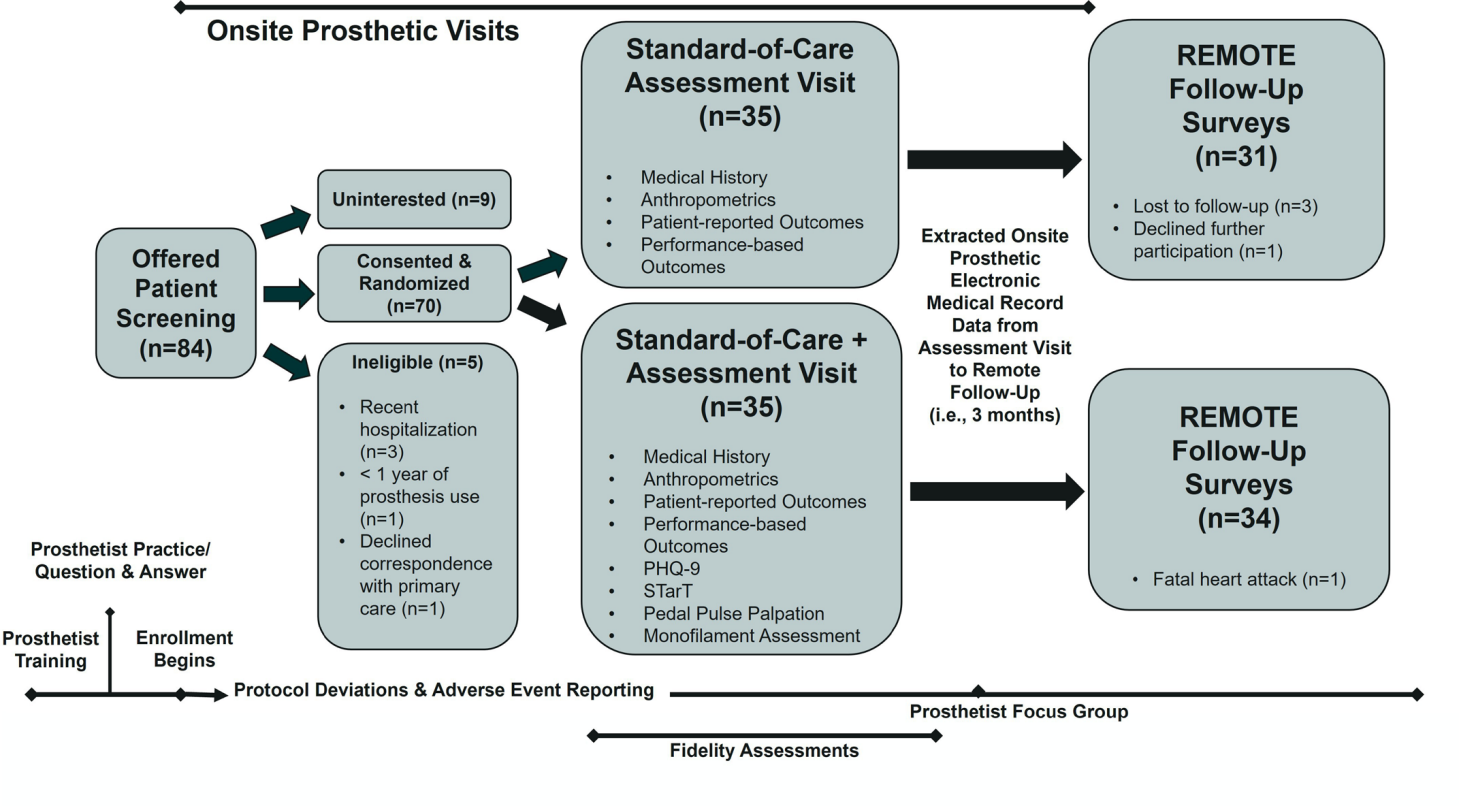
Study flow diagram. PHQ, Patient Health Questionnaire; STarT, STarT Back Screening Tool.

Between June and November of 2022, 70 participants with an LLA were enrolled in the clinical trial following free and informed consent. During a routine prosthetic care assessment visit, individuals were screened, consented and randomised with stratification by amputation level, into one of two arms, that is, SOC or SOC+, by one of four certified prosthetist–orthotists with approximately 38 years of combined experience (range: 1.3–17.7 years). Following participant enrolment, prosthetists opened an envelope with the concealed treatment allocation determined by computer-generated random numbering. Prosthetists underwent 4 hours of clinical trials training, practiced standard assessment evaluations for 1 month, had a brief re-training and question and answer session and then began participant enrolment (figure 1). Training in equipoise, which refers to acceptance by prosthetists that both treatment arms provide a reasonable care approach (particularly since screening, as performed in this study, is not routine in prosthetist clinical practice) was included to mitigate infusion of biases. The study was registered on ClinicalTrials.gov (#NCT05410548) on 6 June 2022.

### Study sample

English-speaking and reading patients, aged ≥18 years, attending routine prosthetic care visits at one of five Independence Prosthetics-Orthotics offices in Delaware or Pennsylvania, with a unilateral transfemoral-level or transtibial-level amputation, who reported using their prosthesis for ≥1 year (regardless of ambulatory status), were recruited for participation. Individuals consented for the research team to access their prosthetic medical records and were excluded if they had contralateral LLA (due to lack of ability to assess non-amputated limb health), impaired cognition precluding informed consent, were hospitalised in the last 3 months, received new or replacement prosthetic componentry in the last 3 months due to the potential confounding influence on subsequent 3-month prosthetic care utilisation and/or were unwilling to have their comorbidity screening results sent to their primary care. Standardised scripts were used during recruitment to reduce patient perceptions of social pressure to participate; to mitigate recruitment bias including gender and racial bias, all patients who presented to the clinic with unilateral transfemoral or transtibial LLA were asked to participate. The offices, which were independent from other medical facilities, were staffed by four certified prosthetists–orthotists (one male; three females), who also consented to research participation.

### Standard of care

Standardised routine care assessments included medical history, anthropometrics (ie, height, weight, residual limb circumference 4 cm proximal to distal end), patient-reported and performance-based outcome measures. Prosthetists obtained average Socket Comfort Score in the past 24 hours, where 0=‘most uncomfortable fit’ and 10=‘most comfortable fit imaginable’^18^ and the Houghton Scale, which evaluates prosthesis use and stability, where scores ≥9 suggest community-ambulatory status.^19^ The Prosthetic Limb Users Survey of Mobility (PLUS-M), which is a reliable and valid 12-item survey, evaluated prosthesis-enabled mobility, where T-scores of 50 represent the mean of a reference sample of >1000 adults with LLA.^20^ Performance-based outcomes, that is, the Timed Up and Go (TUG), Functional Reach and 10-metre walk test (10mWT), were administered with standardised instructions, prosthetist guarding to ensure participant safety and use of an assistive device if needed (except for Functional Reach). For the TUG, participants completed one practice trial and two timed trials at their comfortable pace, which were averaged; participants stood from a standard armchair, walked 3 m, turned and returned to sitting in the chair.^21 22^ Functional Reach was evaluated on the prosthetic side; with the shoulder elevated to 90°, the elbow extended and the hand fisted, participants reached as far forward as possible without losing their balance and distance was recorded and averaged for three trials following two practice trials.^23^ To evaluate gait speeds, participants completed the 10mWT where the central 6 m of a 10 m course were timed allowing for 2 m of acceleration and deceleration at either end of the course; two timed trials were averaged at each speed, that is, self-selected and fast, following a practice trial.^21 22^

### Comorbidity screening

Participants randomised to SOC+ also received screening for major depressive symptoms, suicidal ideation, moderate-to-high risk LBP and lack of pedal pulses and protective sensation in the non-amputated foot. The nine-item Patient Health Questionnaire (PHQ-9) is a widely used reliable and valid measure of depressive symptom severity in the USA, where scores ≥10 have a specificity of 88% for major depression.^24^ The ninth item, when endorsed, is a consistent predictor of suicide attempts and deaths.^25 26^ For individuals with LBP, the six-item STarT Back Screening Tool (STarT) was used to screen for low (≤2) versus medium-to-high risk LBP (≥3); scores ≥3/6 have a specificity of 84% for predicting bothersome and disabling LBP 6 months later.^27^ Non-amputated limb health was evaluated with palpation of the dorsalis pedis and posterior tibial pulses. Absence of both pulses has diagnostic accuracy for PAD as compared with the ‘gold standard’ of ankle brachial index testing.^28^ Protective sensation was evaluated using a 5.07/10 g monofilament applied to the plantar aspect of the great toe and the third and fifth metatarsal heads, while avoiding calluses for up to three trials per site.^29^ A positive screen was an inability to detect ≥1 site^29^; diagnostic accuracy for PN as compared with nerve conduction studies has been previously established.^29^ Participants closed their eyes and the application order and timing of trials was randomised to enhance accuracy and reduce false negative screens.

Screening results were documented in a templated letter on clinic letterhead, reviewed with the participant and faxed to primary care by the prosthetist with the surveys attached. When findings warranted immediate follow-up, that is, major depressive symptoms or suicidal ideation, prosthetists telephoned primary care and documented interactions, as well as provided participants with the national suicide and crisis lifeline telephone number (988).

### Primary outcomes

Via REDCap (Research Electronic Data Capture), all participants were requested to complete the PHQ-9 and the STarT remotely at 3-month follow-up (see figure 1). Participants also completed two satisfaction surveys. The nine-item, Client Satisfaction Inventory (CSI), where higher scores are better, evaluates a patient’s perception of services being relevant, helpful and patient-centred.^30^ The 21-item, Orthotic–Prosthetic Users’ Survey (OPUS) has two subscales, that is, Satisfaction with Device and Satisfaction with Services, and higher scores are better.^31^ Prosthetists were blinded to satisfaction survey results. Participants also reported on healthcare utilisation in the past 3 months, and prosthetic healthcare utilisation was extracted from electronic medical records.

### Statistical and power analyses

Descriptive statistics were computed. χ^2^ and Mann-Whitney U tests were used to evaluate between-group differences; effect sizes were calculated, when appropriate. Regression models were used to test if the relationship between baseline prosthetic visit time and subsequent 3-month prosthetic care utilisation differed between groups. Analyses were conducted using IBM SPSS Statistics (p<0.050) (V.28, Armonk, New York, USA). An a priori power analysis was conducted using G*Power V.3.1.9.7 (Heinrich-Heine-Universität, Düsseldorf, Germany). After accounting for 17% attrition given past research in LLA and prosthetic practice data, that is, six participants per group, with a=0.05, power=0.80 and 35 participants enrolled per group, the study was powered to detect an effect size of 0.66.

### Patient and public involvement statement

Standardised baseline assessments were developed and agreed on by the prosthetic team, which included one individual with an LLA. Performance-based outcomes were selected based on functional mobility challenges expressed by patients with LLA, for example, getting up from a chair, turning, reaching and walking. To evaluate prosthetist acceptability of comorbidity screening, using a semi-structured interview format, the principal investigator conducted a 2-hour focus group in December 2022. Data was recorded by two research team members, summarised by the principal investigator and reviewed for accuracy by two prosthetists.

### Intervention fidelity

Throughout the study, the principal investigator completed manual chart reviews of all onsite assessments (online supplemental figure 1) and provided prosthetists with feedback for improved adherence when <90% of criteria were met. For each prosthetist, the principal investigator also performed one onsite observation of the SOC+ intervention (online supplemental figure 2) to evaluate adherence that could not be obtained from medical charts; feedback was provided following the session.

### Adverse events and protocol deviations

Adverse events, observed by the prosthetist or reported by the participant, were defined as any unfavourable or untoward medical occurrence in a participant, including any abnormal sign, symptom or disease associated with research involvement, whether or not the event was considered related to research participation. Protocol deviations were defined as any departure from the study procedure or treatment plan as specified in the IRB-approved protocol. Prosthetists completed a possible adverse event and/or protocol deviation form, which was adjudicated by the principal investigator and reported, when required, to the local IRB.

## Results

### Study sample

There were no significant between-group differences in participant demographics or data obtained from standardised clinical evaluations (table 1; p>0.050). The sample was 68.6% male and had a median age of 60.0 years. Of the participants, 71.4% had a transtibial-level amputation and the median time since amputation was 7.9 years. Per the Houghton Scale, participants were largely independent community ambulators.^19^ The PLUS-M suggested prosthesis-enabled mobility was around the 67th percentile as compared with peers with unilateral LLA. Performance-based outcome measures, that is, TUG and 10mWT, suggested the sample would be classified predominantly at higher functional mobility levels.^32^

**Table 1:** Participants characteristics at baseline assessment visit

	SOC (n=35)	SOC+ (n=35)
Demographics		
Level of amputation, transtibial	25 (71.4)	25 (71.4)
n	35	35
Sex, male*	22 (62.9)	26 (74.3)
n	35	35
Race, Caucasian	25 (71.4)	28 (80.0)
n	35	35
Ethnicity, non-Hispanic/Latino	33 (94.3)	32 (94.1)
n	35	34
Cause of amputation		
PVD/diabetes	9 (25.7)	12 (34.3)
Infection	5 (14.3)	8 (22.9)
Trauma	11 (31.4)	4 (11.4)
Congenital	1 (2.9)	3 (8.6)
Other	7 (20.0)	6 (17.1)
Multiple	2 (5.7)	2 (5.7)
n	35	35
K-level		
K2	5 (14.3)	4 (11.4)
K3	14 (40.0)	12 (34.3)
K4	16 (45.7)	19 (54.3)
n	35	35
Time since amputation, years	9.3 (4.6, 16.2)	6.4 (3.0, 25.2)
n	35	35
Age, years	60.0 (53.0, 65.0)	61.0 (55.0, 71.0)
n	35	35
Standardised clinical evaluation		
Anthropometrics		
Height, cm	174.6 (164.5, 180.1)	177.8 (168.9, 182.9)
n	34	35
Weight with prosthesis, kg	88.4 (71.0, 109.9)	89.2 (69.4, 104.6)
n	34	32
Weight of prosthesis and liner, kg	3.0 (2.5, 4.3)	3.4 (2.8, 4.8)
n	34	35
Residual limb circumference, cm	28.0 (24.0, 32.0)	30.0 (24.0, 33.0)
n	35	35
Patient-reported outcome measures		
Average SCS, 0–10	8.0 (7.0, 9.0)	8 (5.0, 10.0)
n	35	34
Houghton Scale, 0–12	10 (9, 11)	10 (8, 12)
n	35	35
PLUS-M, T-score	53.6 (49.1, 61.0)	56.3 (48.4, 61.0)
n	35	35
Performance-based outcome measures		
Assistive device used		
None	27 (81.8)	30 (85.7)
Cane	2 (6.1)	0 (0)
Crutches	2 (6.1)	0 (0)
Walker	2 (6.1)	5 (14.3)
n	33	35
TUG, sec§	10.48 (8.42, 13.59)	9.36 (8.18, 12.71)
n	35	34
Functional Reach-prosthetic side, cm†	22.9 (18.6, 27.8)	22.5 (18.2, 29.4)
n	35	34
10-metre walk test-SS, m/s‡	1.10 (0.87, 1.28)	1.13 (0.90, 1.30)
n	35	33
10-metre walk test-F, m/s‡	1.36 (1.04, 1.59)	1.37 (1.12, 1.59)
n	35	33
Comorbidity screening		
Pulse palpation, n	–	35
Absence of ≥1 pulse		12 (34.3)
Absence of both pulses		5 (14.3)
Monofilament assessment, n	–	35
Lack of protective sensation		14 (40.0)
PHQ-9, n	–	35
Major depressive symptoms		3 (8.6)
Endorsement of suicidal ideation		3 (8.6)
STarT Back Screening Tool, n	–	16
Moderate-to-high risk LBP		3 (18.8)
Overall screening results, n		35
≥1 concerning finding ≥2 concerning findings ≥3 concerning findings		21 (60.0) 11 (31.4) 2 (5.7)

Data is presented as n (% of sample) or median (25th, 75th percentile).

* Self-reported sex options were male, female or prefer not to say.

† Average of three trials without assistive device; removed participant that did not complete three trials.

‡ Average of two trials; removed two participants that did not complete per-protocol.

§ Average of two trials; removed participant that did not complete two trials.

cm, centimetres; F, fast; kg, kilograms; LBP, low back pain; m/s, metres/second; PHQ-9, Patient Health Questionnaire-9 item; PLUS-M, Prosthetic Limb Users Survey of Mobility; PVD, peripheral arterial disease; SCS, Socket Comfort Score; sec, seconds; SOC, standard-of-care; SOC+, standard-of-care + comorbidity screening; SS, self-selected; STarT, STarT Back Screening Tool; TUG, Timed Up and Go.

### Comorbidities

Within the SOC+ treatment arm, 34.3% of participants had at least one non-palpable pulse; of these, five participants had non-amputated side, non-palpable dorsalis pedis and posterior tibial pulses suggesting PAD (table 1). Within the SOC+ arm, 40.0% lacked protective sensation in ≥1 location on the non-amputated side, suggesting PN and compromised limb health.

Major depressive symptoms were present in three SOC+ individuals, of whom two also reported suicidal ideation (table 1). A third participant reported only suicidal ideation at baseline. Via remote 3-month follow-up, three more participants reported major depressive symptoms and two more reported suicidal ideation. Thus, a total of nine individuals (11.4%) reported mental distress during the 3-month observation period. Of the 12 communications with primary care, 5 telephone calls were elevated to a nurse or higher; prosthetists deemed all interactions with primary care, neither ‘positive’ nor ‘negative’, but rather ‘neutral’.

Over the 3-month observation window, 44 of 70 participants (62.8%) self-reported LBP on their medical history form, of which 10 (14.3%) had moderate-to-high risk LBP. In the SOC+ arm, at baseline 3 of the 16 with current LBP were at moderate-to-high risk for persistent, disabling LBP (table 1); one continued to have moderate-to-high risk at 3-month follow-up. Two additional cases of moderate-to-high risk LBP were identified in the SOC+ at 3 months. In the SOC group, five individuals were identified as moderate-to-high risk for persistent, disabling LBP via the remote 3-month follow-up.

### Care utilisation and satisfaction

There was no significant difference in baseline visit time between the SOC and SOC+ arms, that is, 70 min (25th percentile: 55, 75th percentile: 78) and 76 min (25th percentile: 58, 75th percentile: 89), respectively (p=0.249). Table 2 shows no significant between-group difference in prosthetic care utilisation after the onsite assessment visit as extracted from the prosthetic electronic medical record nor 3-month follow-up satisfaction surveys (p>0.050). Of the 70 participants, 54.3% had ≥1 prosthetic follow-up visit within the 3-month observation window and the median visit length was 36 min (table 2). While there was no difference in total appointment time between groups, there was a difference in the relationship between baseline onsite visit time and total appointment time between groups. The model with the interaction was significantly improved, ΔR^2^=0.131, _adj_R^2^=0.315, *F*(1,34)=7.07, p=0.012. In SOC, there was a strong positive relationship (b=3.33, t(34)=4.18, p=0.002) showing that longer baseline visits lead to longer subsequent appointment times; this did not hold for SOC+, which had no relationship (b=−0.061, t(34)=0.06, p=0.952). This finding suggests that even though SOC+ is not initially quicker, it may help reduce time in subsequent follow-up appointments for those with longer baseline visits.

**Table 2:** Care utilisation and satisfaction between treatment arms

	SOC (n=35)	SOC+ (n=35)	P value
EMR data			
Visits baseline to 3 months	17 (48.6)	21 (60.0)	0.337
One visit	7 (41.2)	9 (42.9)	0.410
Two visits Three or more visits	3 (17.6) 7 (41.2)	7 (33.3) 5 (23.8)	
Average visit length, min	39 (26, 58)	33 (25, 53)	0.450
Cumulative care, min	77 (36, 169)	51 (33, 119)	0.352
Outcome measures			
CSI, 0–100%*	100 (96.8, 100)	100 (96.3, 100)	0.829
n	28	31	
OPUS: device, 11–55*	40 (33, 46)	43 (38, 49)	0.178
n	27	32	
OPUS: service, 10–50*	47 (40, 50)	49 (44, 50)	0.135
n	30	33	

Data is presented as median (25th, 75th percentile) or n (% of sample).

* Some participants did not fully complete the surveys rendering the survey not scorable.

CSI, Client Satisfaction Inventory; EMR, electronic medical record; min, minutes; OPUS, Orthotic–Prosthetic Users Survey; SOC+, standard-of-care + comorbidity screening; SOC, standard-of-care.

**Figure 2:**
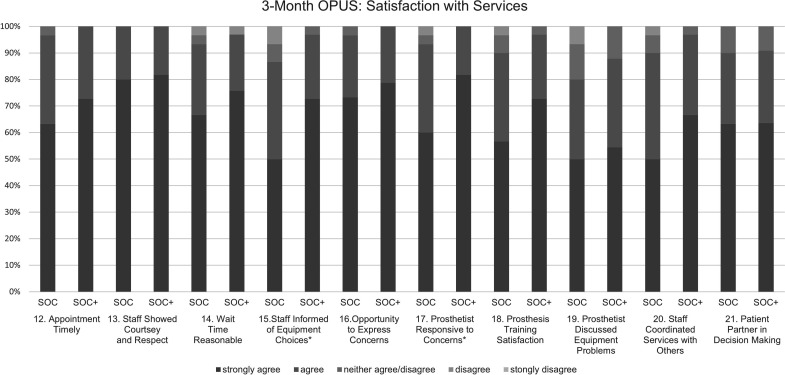
3-month OPUS: Satisfaction with Services subscale by treatment arm. There were significant between-group differences (*) related to item 15, which assesses the degree to which participants feel informed of equipment choices (Z=−1.990, p=0.047, r=0.251) and item 17, which assesses responsiveness of the prosthetist to participant concerns and questions (Z=−1.994, p=0.046, r=0.251), favouring the SOC+ treatment arm. OPUS, Orthotic Prosthetic Users Survey; SOC, standard-of-care; SOC+, standard-of-care + comorbidity screening.

For the CSI, for every item, >90% of participants endorsed ‘all of the time’ or ‘most of the time’ suggesting high general care satisfaction. For the OPUS Satisfaction with Prosthesis Subscale, there were significant between-group differences for prosthesis fit, with 78.8% of the SOC+ group reporting that their ‘prosthesis fit well’, as compared with 60.0% in SOC (Z=−2.045, p=0.041, r=0.258), but there were no other significant between-group differences (p>0.050). Notably, only ~40% of participants reported they could afford to purchase, maintain, repair or replace their prosthesis. Further, only 50% reported their prosthesis was pain-free to wear. For the OPUS Satisfaction with Services (figure 2), there were significant between-group differences for item 17, which assesses prosthetist responsiveness to concerns; 100% of SOC+ individuals ‘agreed’ or ‘strongly agreed’ that their prosthetist was responsive, while 93.7% of SOC ‘agreed’ or ‘strongly agreed’ (Z=−1.994, p=0.046, r=0.251).

### Prosthetist perspective

All four prosthetists participated in the focus group (see online supplemental figure 3). Prosthetists did not use the PHQ-9, STarT, pulse palpation or Functional Reach prior to the trial. Most prosthetists acknowledged learning monofilament testing in their orthotics and prosthetics training programme, although three of four reported not assessing protective sensation routinely. Prosthetists agreed monofilament testing was valuable in that it reminded them to look at the non-amputated foot and facilitated a conversation about general limb health. Pedal pulse palpation was found to be challenging, with prosthetists expressing concerns regarding potential bias given the patient’s self-reported medical history (eg, PAD) and lack of confidence in correctly interpreting pulse presence. Persistent prosthetist discomfort with depression, suicidal ideation and medium-to-high risk LBP screening was expressed, with suggestions for enhanced training. Prosthetists liked that the results letter helped to facilitate explanation of the findings to the patient. The group agreed screening helped to ‘show they were part of the healthcare team, and they care’. Prosthetists suggested the following screens for future consideration: blood pressure (with automated cuff), heartrate (with pulse oximeter) and standardised skin/wound assessment.

### Intervention fidelity

Per 70 chart reviews, intervention fidelity was 95.7% and 95.9% in the SOC and SOC+ arms, respectively. Observations of SOC+ interventions among the four prosthetists indicated 99% fidelity.

### Adverse events and protocol deviations

Two adverse events were recorded: shortness of breath during performance testing, which was related to study participation but resolved during the visit, and a fatal heart attack during the 3-month follow-up window, unrelated to study participation. Eight protocol deviations were reported: five due to performance testing modification, two due to modified methods to obtain data and one due to missing chart data, suggesting the need for flexibility in data obtainment to reduce data missingness.

## Discussion

Most studies on comorbidity in LLA are longitudinal cohort studies. This is the first randomised, controlled clinical trial evaluating comorbidity screening potential benefits, acceptability and feasibility during routine prosthetic care visits. The study provides a framework for future clinical trials conducted during prosthetic clinical care. Our primary hypothesis that SOC+ would result in greater patient satisfaction at 3-month (remote) follow-up and reduce prosthetic care minutes over the subsequent 3 months was not supported, although notably satisfaction did not decrease with screening. Further, prosthetists’ feedback suggests comorbidity screening for protective sensation is valuable for facilitating a conversation regarding non-amputated limb health, while screening for major depressive symptoms, suicidal ideation and medium-to-high risk LBP might be adopted in routine practice with increased prosthetist training to enhance prosthetist comfort. Primary care response to prosthetist screening and referral for major depressive symptoms and suicidal ideation may help to mitigate prosthetists’ fears that psychological screening might be considered outside the prosthetists’ scope of practice. Further, patients receiving comorbidity screening may feel that the prosthetist is more responsive to concerns. And the addition of comorbidity screening did not significantly increase patient care time, suggesting it may be feasible during prosthetic visits.

Among a large, heterogeneous group of persons post-amputation, Pezzin *et al* found an average of three comorbidities per person; greater comorbidity burden was associated with less prosthesis use.^16^ Of our 35 participants with LLA who underwent comorbidity screening during the onsite visit, 21 had ≥1 concerning screening finding; 11 had ≥2 concerning screening findings and 2 had ≥3 concerning findings. The most common positive finding was lack of protective sensation, occurring in 14 of 35 participants. Of these 14 participants, 1 reported PN on their medical history, while 5 reported ‘nerve damage in [their] feet’; use of common vernacular has been shown to enhance self-report.^33^ Non-palpable pulse(s), suggesting compromised blood flow, occurred in 13 of 35 participants, with 8 participants having either an absent dorsalis pedis or posterior tibial pulse and 5 participants having absence of both non-amputated-side pedal pulses. Of these 13 participants, only 3 reported PAD or peripheral vascular disease, while 6 reported ‘poor blood flow to [their] legs/feet’. Our findings align with prior research demonstrating >50% of adults with LLA who have clinical signs indicative of PN and/or PAD do not self-report these medical conditions.^34^ Thus, hands-on clinical assessment of non-amputated limb health is critical in this patient population.

In the general population, comorbidity screening positively influences health-seeking behaviours.^35^ Among the SOC+ group, we found the majority with positive screens received care from another provider within the 3-month follow-up. For example, 9 of 14 participants with suspected PN sought care (n=5 podiatrist, n=2 neurologist, n=1 endocrinologist, n=1 multiple providers), while 9 out of 12 received follow-up care for suspected PAD (n=5 vascular specialist, n=3 podiatrist, n=1 multiple providers). Nevertheless, prosthetists remained sceptical regarding pulse palpation in clinical practice. Concerns are validated by research questioning the accuracy of dorsalis pedis and posterior tibial pulse palpation in bustling clinical practice, and particularly by novice clinicians.^36^,^38^ As such, assessment of pedal pulses might be better left to medical specialists (eg, vascular surgeons) or supplemented with diagnostic technology to enhance accuracy.^38^

In primary care settings, universal depression screening is recommended.^39^ Practitioners may be hesitant to discuss depression, but a recent study found patients are moderately comfortable with discussing depression with their care provider, especially when providers clearly articulate the relevance.^40^ Given that depression predicts long-term patient satisfaction after lower-extremity injury^41^ and is part of the grief cycle following amputation, normalisation of depression is recommended, as is patient education regarding the relevance of depression screening to prosthetic care. For example, depression may negatively impact post-amputation adjustment and prosthesis satisfaction.^42^ Perhaps the greatest hurdle to universal depression screening by prosthetists is enhancing comfort through case-based training that equips prosthetists with discipline-specific rationales for depression screening and clear follow-up procedures.

Prosthetist training in depression screening pairs well with suicidal ideation screening since suicidal ideation may occur concurrently with depressive symptoms but receives less attention (and is included on the PHQ-9). Prosthetist discomfort with suicidal ideation screening and referral is not unexpected given a recent study of healthcare providers found one-third or less reported feeling knowledgeable about assessing suicide risk and confident in appropriately responding.^43^ Provider comfort in responding to suicidal ideation may be enhanced with clear referral procedures and communication training that includes standardised patient cases and, when possible, the use of medical actors.^44^

A 2019 review found STarT was the best screening tool available for identification of patients at risk for persistent, disabling LBP; specifically, STarT helps identify those who would benefit from physical therapy interventions addressing biopsychosocial aspects of the LBP experience.^45^ Further, the National Institute for Health and Care Excellence recommends STarT to inform shared decision-making with the patient as it relates to new issues of LBP.^46^ Prosthetists admitted they were unlikely to continue using STarT without a better action plan for implementation when screens were positive. In reflection, we believe the pre-trial training, which only covered STarT scoring and interpretation, was insufficient to evoke practice change incorporating STarT.

High patient satisfaction with services provided by prosthetists aligns with prior studies.^16 17^ At the 3-month follow-up, maximal scores were present in 38.1% and 66.1% of participants for OPUS Services subscale and CSI, respectively, which may have negated the ability to detect between-group differences. Given the unanticipated ceiling effect, alternatives for evaluating prosthetic service satisfaction in future clinical trials are recommended. Of note, participants within the SOC+ arm rated higher agreement with the statement that the prosthetist was responsive to their concerns. Findings might be explained by participant recall of discussions during the patient education component post-screening. While it is unlikely that selected comorbidity screens covered all patient concerns, perhaps such dialogue prompted discussion of other concerns. Supplementing future trials with participant focus groups may elucidate the factors associated with perceived prosthetist responsiveness.

More than 50% of our participants had at least one additional prosthetic visit within the 3-month observation window, with a median visit length of 36 min. While SOC and SOC+ did not significantly differ in their average follow-up appointment time, the significant interaction of initial visit time by group suggested that SOC+ may help reduce subsequent follow-up appointment times for patients with longer prior appointments. For example, an individual classified as >low risk LBP may require treatment addressing both biological and psychological aspects of their pain experience and may be referred for comprehensive physical therapy that addresses thoughts and behaviours related to LBP in addition to biological factors. During subsequent prosthetic visits, patients may be less likely to erroneously contribute their LBP to biological factors, like prosthetic alignment, when psychological factors may be greater contributors to their LBP experience. Future studies may confirm these findings, supporting a long-term business benefit to prosthetist comorbidity screening.

Pezzin *et al* reported an average of 9±11 visits to prosthetists in a given year among 935 persons with limb amputation; only 12% of respondents reported no prosthetist visits.^16^ Dissatisfaction with prosthesis comfort has been previously reported by up to 33% of individuals post-amputation.^16^ Only 50% of our participants reported their prosthesis was pain-free to wear despite being longer-term prosthesis users. Collectively, data suggest prosthetic visits for optimising fit and function are common, yet there is little research regarding the cost of such visits,^47^ and whether additional fee-for-service billing codes for prosthetists might be supported.^48^

### Study strengths and limitations

Procedural fidelity was excellent as suggested by onsite observations and medical chart review. Adverse events and protocol deviations were few. Yet, future studies may consider alternative satisfaction surveys (ideally with validation in adults post-amputation) and extend follow-up windows beyond 3 months to evaluate the impact of comorbidity screening on relevant health outcomes, for example, limb wound development and subsequent amputation. Results are from a single prosthetic practice and generalisable to adults with a unilateral transfemoral or transtibial amputation who receive prosthetic care; external generalisability may be enhanced with a multisite trial that includes a more heterogeneous LLA population (eg, lower functional mobility levels) and replication in practice settings that see both individuals who do and do not use a prosthesis. Finally, sample size precluded evaluation of patient-specific factors, for example, sex, associated with care-seeking behaviours and given lack of integrated healthcare records for our participants, self-reported healthcare utilisation was unable to be verified with electronic medical records

## Conclusions

This novel clinical trial finds comorbidity screening by prosthetists may be feasible in that it did not significantly increase visit length. Assessment of protective sensation may facilitate conversation with the patient regarding limb health and might be more readily adopted by prosthetists as compared with screening for depressive symptoms, suicidal ideation and medium-to-high risk LBP, which would require significantly more training to overcome barriers to adoption in clinical practice. Acceptability of prosthetists providing comorbidity screening was evidenced, and participants who received comorbidity screening were more likely to agree that their prosthetist was responsive to their concerns.

## Supplementary material

10.1136/bmjopen-2025-108623 online supplemental figure 1

10.1136/bmjopen-2025-108623 online supplemental figure 2

10.1136/bmjopen-2025-108623 online supplemental figure 3

## Data Availability

Data are available upon reasonable request.
