# Supplementary material for: Prosthetist screening for comorbidity during routine care visits: a randomised controlled clinical trial evaluating benefits, acceptability and feasibility
Source: BMJ Open. 2026 Feb 27;16(2):e108623. doi: 10.1136/bmjopen-2025-108623 (PMC12959007; doi:10.1136/bmjopen-2025-108623)
Supplement: online supplemental figure 1 [file bmjopen-16-2-s001.pdf]

APPENDIX: FIGURE 1. CHART REVIEW FIDELITY CHECKLIST FOR CLINICAL EVALUATION		
**Site**		
**Date**		
**Participant ID**		
**Prosthetist ID**		
	**AGREE (1)**	**DISAGREE (0)**
**STANDARD OF CARE (all participants)**		
1. Documented weight of participant wearing prosthesis in X.X pounds in medical chart.		
2. Documented weight of prosthetic system (limb+socks+liner) in X.X pounds in medical chart.		
3. Documented residual limb size in XX.X centimeters in medical chart.		
4. PLUS-M T-score AND percentile documented in medical chart.		
5. Documented average self-selected in X.XX m/sec in medical chart.		
6. Documented average fast gait speed in X.XX m/sec in medical chart.		
7. Recorded average time for Timed Up and Go in XX.XX sec in medical chart.		
8. Recorded average distance for the Functional Reach Test in X.X cm in medical chart.		
9. No missing data for minimal data set in medical chart (without protocol deviation form).		
10. Demographics, SF-20, Houghton, SCS, and PLUS-M scanned and attached to medical chart visit.		
**PLUS SCREENING (only screening arm participants)**		
11. Documented pedal pulse integrity for both dorsalis pedis AND posterior tibialis in medical chart.		
12. Documented presence/absence of sensation at 3/3 ‘sound limb’ sites in medical chart.		
13. PHQ-9 correctly scored/interpreted for major depression (per patient/provider handout).*		
14. PHQ-9 suicidal ideation question appropriately interpreted (per patient/provider handout).*		
15. STaRT Back Tool correctly scored/interpreted (per patient/provider handout), if back pain.*		
16. Correctly marked pulse integrity data on patient/provider handout.*		
17. Correctly marked sensation data on patient/provider handout.*		
18. Provider documented in medical chart that they gave participant a written copy of the results.*		
19. Patient/provider handout, PHQ-9, AND STaRT Back (prn) scanned & attached to medical chart.*		
20. Medical chart indicates provider was faxed a copy of the patient/provider handout & phone call made, if applicable, for major depressive symptoms and/or suicidal ideation.*		
SCORING **TOTAL**		
1 point for each AGREE; 0 points for each DISAGREE (Target>90% for both treatment arms)<br>*Maximal point value for STANDARD OF CARE = 10 (if <9, speak with prosthetist, review relevant procedures)*<br>*Maximal point value for STANDARD OF CARE PLUS SCREENING = 20 (if <18, speak with prosthetist, review relevant procedures)*<br>**But, if any critical items are DISAGREE, revisit policies/procedures with prosthetist.*		
If score <90%, was it expected given the context of the appointment? YES or NO (circle one) If expected, give reason why:		

Abbreviations: PLUS-M, Prosthetic Limb Users Survey of Mobility; PHQ-9, Patient Health Questionnaire 9-item; prn, as needed; SCS, Socket Comfort Score.
