# Supplementary material for: Prosthetist screening for comorbidity during routine care visits: a randomised controlled clinical trial evaluating benefits, acceptability and feasibility
Source: BMJ Open. 2026 Feb 27;16(2):e108623. doi: 10.1136/bmjopen-2025-108623 (PMC12959007; doi:10.1136/bmjopen-2025-108623)
Supplement: online supplemental figure 2 [file bmjopen-16-2-s002.pdf]

APPENDIX: FIGURE 2. ON-SITE FIDELITY CHECKLIST FOR CLINICAL EVALUATION		
**Site**		
**Date**		
**Participant ID**		
**Prosthetist ID**		
	**AGREE (1)**	**DISAGREE (0)**
**STANDARD OF CARE (all participants)**		
1. Collected weight of participant wearing prosthesis.		
2. Collected weight of prosthetic system (limb+socks+liner).		
3. Measured residual limb size 4cm from distal end.		
4. Completed 6 trials of the 10-meter Walk Test (i.e., 3 self-selected, 3 fast).		
5. Correctly calculated gait speed.		
6. Provided standardized verbal prompts for 10-meter walk test for each condition.		
7. Completed a practice and 2 timed trials for the Timed Up and Go (i.e., 3 trials total).		
8. Provided appropriate verbal prompt for Timed Up and Go.		
9. Completed 2 practice trials and 3/3 recorded trials for the Functional Reach Test (i.e., 5 trials total).		
10. Properly guarded patient to reduce fall risk during the Functional Reach Test.		
11. Practitioner was NOT present during administration of satisfaction questionnaires.		
12. Provider handed patient envelope to deposit questionnaires to reduce potential for bias.		
**PLUS SCREENING (only screening arm participants)**		
**DEPRESSIVE SYMPTOMS**		
13. Administered the PHQ-9.		
14. Appropriately scored and interpreted the PHQ-9.		
**PERIPHERAL ARTERIAL DISEASE**		
15. Assessed pedal pulse integrity for both dorsalis pedis AND posterior tibialis.		
16. Assessment of presence/absence agrees with observer.		
**PROTECTIVE SENSATION**		
17. Practitioner performed practice on forearm with the participant's eyes opened.		
18. Practitioner applied monofilament with appropriate bend and with appropriate duration (1 sec).		
19. Practitioner performed monofilament testing on 3/3 sound limb study sites.		
20. Practitioner had participant close their eyes for monofilament testing.		
21. Practitioner applied up to 3 touches per site, as necessary, prior to ruling test as 'absent'.		
**>LOW RISK FOR PERSISTENT LOW BACK PAIN (if participant endorsed low back pain)**		
22. Practitioner administered the STarT Back Tool.		
23. Practitioner appropriately interpreted the STarT Back Tool information.		
24. Gave participant a written copy of the results.		
25. Discussed screening findings and any 'next steps' with the participant.		
26. Sent written results to the participant's primary care provider.		
SCORING **TOTAL**		
1 point for each AGREE; 0 points for each DISAGREE (Target>80%) *Maximal point value for STANDARD OF CARE PLUS SCREENING = 26 (if <24, speak with prosthetist, review relevant procedures)*		
If score <80%, was it expected given the context of the appointment? YES or NO (circle one) *If expected, give reason why:*		

Abbreviations: PHQ-9, Patient Health Questionnaire 9-item.
