# Supplementary material for: Prosthetist screening for comorbidity during routine care visits: a randomised controlled clinical trial evaluating benefits, acceptability and feasibility
Source: BMJ Open. 2026 Feb 27;16(2):e108623. doi: 10.1136/bmjopen-2025-108623 (PMC12959007; doi:10.1136/bmjopen-2025-108623)
Supplement: online supplemental figure 3 [file bmjopen-16-2-s003.pdf]

SUPPLEMENTAL FIGURE 3. PROSTHETIST FOCUS GROUP SCRIPT

Welcome, thank you for being here today. The purpose of this gathering is to get your feedback about what barriers and facilitators exist for prosthetists, such as yourselves, to perform screenings for common comorbidities among their patients with lower-limb loss. Specifically, we want to understand which assessment tools worked best for you, why, and how to encourage use of these tools among prosthetists. You have a better understanding of what works than we do, which is why we are talking with you.

I am ____________ and I will be the moderator in today's discussion. The format we are using is a focus group. A focus group is a conversation that focuses on specific questions in a safe and confidential environment. I will guide the conversation by asking questions that each of you can respond to. There are no right or wrong answers to these questions. Just be honest. If you wish, you can also respond to each other's comments, like you would in an ordinary conversation. It is my job to make sure that everyone here gets to participate and that we stay on track. _______ is here to record and summarize your comments.

Before we get started, I want to let you know two things. First, the information we learn today will be compiled and may be utilized in future publications related to the study you assisted with data collection for. Publications may include key themes and/or quotes, but all comments will be de-identified. Secondly, you do not have to answer any questions that you do not feel comfortable with. This focus group today is anonymous and confidential. "Anonymous" means that we will not be using your names and you will not be identified in any publication. However, because this is a small study with only a single Prosthetic and Orthotic company, i.e., Independence Prosthetics-Orthotics, Inc., group anonymity cannot be guaranteed. "Confidential" means that what we say in this room should not be repeated outside of this room. Obviously, I cannot control what you do when you leave, but I ask each of you to respect each other's privacy and not tell anyone what was said by others here today. Although we hope everyone here honors this confidentiality, please remember that what you say here today could be repeated by another focus group member or appear in summative feedback related to this study. So please, do not say anything that you absolutely need to keep private.

Does anyone have any questions before we begin?

Example Focus Group Questions:

<u>Opening Question:</u> Prior to this study, which clinical assessment tools used in this study did you use in your own clinical practice?

Follow-up: Were there any assessment tools you were unfamiliar with prior to the study?

<u>Key Question:</u> What do you feel would be the biggest barriers to you continuing to utilize these assessment tools in your clinical practice?

<u>Key Question:</u> What would be some ways you feel would facilitate use of these tools among other prosthetic practitioners?

<u>Ending Question:</u> How likely are you to use the screening tools from this study in your future clinical practice?

Follow-ups: What do you like about the tools you plan to use? What is discouraging you from using the other(s)?
