# Supplementary material for: Prosthetist screening for comorbidity during routine care visits: a randomised controlled clinical trial evaluating benefits, acceptability and feasibility
Source: BMJ Open. 2026 Feb 27;16(2):e108623. doi: 10.1136/bmjopen-2025-108623 (PMC12959007; doi:10.1136/bmjopen-2025-108623)
Supplement: Supplementary file 4 [file bmjopen-2025-108623.reviewer_comments.pdf]

PEER REVIEW HISTORY

BMJ Open publishes all reviews undertaken for accepted manuscripts. Reviewers are asked to complete a checklist review form and are provided with free text boxes to elaborate on their assessment. These free text comments are reproduced below.

ARTICLE DETAILS

Title (Provisional)

Prosthetist Screening for Comorbidity during Routine Care Visits: A Randomized Controlled Clinical Trial Evaluating Benefits, Acceptability, and Feasibility

Authors

Sions, Jaclyn Megan; Stauffer, Samantha J; Horne, John R; Pohlig, R

VERSION 1 - REVIEW

**Reviewer**	**1**
**Name**	**Landy, David**
**Affiliation**	**University of Kentucky, Department of Orthopaedic Surgery**
**Date**	**03-Oct-2025**
**COI**	**None**

Thanks for sharing this work. Seems to have been really well done and is a great step moving forward. Minor point but I view the value here not in that satisfaction increased with the intervention but rather that it did not decrease. I think the real value long-term here would be showing this could actually improve clinical outcomes. So the main value of this pilot is showing this intervention is feasible.

1. The outcomes, satisfaction and time, fit with a pilot study in that they are continuous so easy to power, easy to measure, and don't require extended follow-up. With that said, it was not clear to me why these outcomes would necessarily be positive. I could imagine value in such a program and improving patient outcomes even if it did not save time or improve initial satisfaction. With that said, I do appreciate that satisfaction or rather lack of dissatisfaction and reasonable time add may be important to prostheses to get them to implement this.

On that note, I'd mention that even though there was not a statistically significant difference in the times of the baseline visits (70 vs 76min), there likely is a difference. I think many clinicians will be surprised how long these visits are so adding 6min seems not clinically significant in this context. I think the modeling of predicting the times of other visits is complicated and may be straying a bit far.

2. Please remove P values and hypothesis testing from Table 1 and the results text. While I appreciate you may have included these expecting a reviewer to ask for them, they are not ideal. If interested in discussion of this topic, please see https://pubmed.ncbi.nlm.nih.gov/35230980/ and https://pubmed.ncbi.nlm.nih.gov/37392076/.

**Reviewer**	**2**
**Name**	**Vanicek, Natalie**
**Affiliation**	**University of Hull, Sport, Health and Exercise Science**
**Date**	**21-Oct-2025**
**COI**	**None**

bmjopen-2025-108623

This pilot RCT explores the feasibility and acceptability of comorbidity screening as part of routine care by prosthetists compared to usual care. The rationale is sound and would be of interest to professionals working within prosthetics therapies internationally. The authors cite that 87% of patients with a LLA are cared for by prosthetists, but what about non-prosthesis users. They are unlikely to see prosthetists, and monitoring comorbidities in this group would be equally important, if not more important. Should there be standardised screening for comorbidities across all amputee patients? Please address this last point in the Discussion.

Introduction

Lines 63-64: when after amputation do depression and suicidal ideation exceed the general population? Does time since amputation affect these aspects of mental health?

Lines 72-73: why has the move to electronic medical records impacted comorbidity screening negatively?

Lines 87-88: I believe this statement is related to the Netherlands (reference 17) and this should be made clear as healthcare models vary greatly across countries. Conversely, reference 18 relates to Iran so the authors should be cautious when making statements that may not be generalisable across countries’ economic status vs healthcare models.

Methods

Line 116: why was prosthesis use of more than 1 year an inclusion criteria? Wouldn’t monitoring comorbidities (e.g. depression) as early as possible be important for new amputees? Why were bilateral amputees excluded?

Lines 137-139: how was safety (i.e. falls prevention) ensured during the functional reach test?

Lines 141-142: did you monitor walking aid usage during the 10-metre walk test? If so, can you report it? If not, why not?

Line 163: how was participant anonymity (and/or protection of personal identifiable details) ensured if findings were faxed to primary care centres? Fax seems a very outdated and insecure way of sharing sensitive data.

Line 170: has the client satisfaction inventory been validated for use in healthcare settings and/or with prosthesis users?

Lines 173-175: was patient-reported healthcare resource use cross-checked with the medical record data?

Line 183: what was the rationale for 17% attrition that was anticipated? What outcome measure was used to power the sample size calculation?

Lines 188-189: did patients inform the choice of outcomes? Were they consulted in the design of the trial?

Lines 191-192: why weren't focus groups done with patients as well as prosthetists to ascertain acceptability of the comorbidity screening?

Can you describe the prosthetic site/practice that was used for this study. It's size, geographical catchment area, number (and type) of healthcare professionals working there, healthcare model, etc …..

Results

Line 219: it seems strange that the sample had a higher functional mobility level when the majority of amputees tend to be older and have had their amputation related to disease and therefore have lower mobility levels.

Throughout this section, please spell out numbers when twelve or less. E.g. Three SOC+ individuals …..

Lines 278-279 and lines 306-308: prosthetists reported feeling uncomfortable dealing with patient-reported feelings of depression and/or suicide. Did the prosthetics centres have "in-house" counsellors and/or psychologists? This could make the prosthetists more comfortable screening for these mental health issues if experts were "at hand" to help patients deal with them.

Line 289-290: why was shortness of breath (in itself) an adverse event? Did it lead to further complications?

Discussion

Lines 315-326: this seems like reporting of results. Could the numerical results be summarised in one of the tables (and then referred to here) so that the narrative is more critically reflective in the Discussion section.

According to the functional performance-based outcome data, your sample was high functioning. Did you not collect data regarding K-levels? Can you report it? Can you comment further on the implications of sampling a high functioning group of patients vs. mostly K1-K2 users? You touch on this lightly in Line 219, but it needs addressing in the Discussion too.

Figure 1 should be titled as a CONSORT flowchart

**Reviewer**	**3**
**Name**	**Clemens, Sheila M.**
**Affiliation**	**Florida International University**
**Date**	**02-Nov-2025**
**COI**	**None**

This study addresses an important area of clinical care that is understudied in the LLA population and offers the foundation for future research to improve care of patients. Tracking comorbidities over time is an important, understudied area of LLA research and this study offers information to fill gaps in knowledge. Additionally, this study provide a basis for future clinically implementable assessments to potentially enhance care of patients with LLA. However, the Methods of the study to include baseline and follow up visits are unclear and would benefit from significant revision to improve reader understanding. Some of the most interesting findings resulted from the CPO interviews and realistic clinical implications for the screenings. Furthermore, the manuscript frequently alludes to suicide an important comorbidity, and this topic is understudied in amputation research. However, the study rationale doesn't reflect on why it's important to screen for suicide risk in the LLA population in particular, or the urgency by which it should be addressed. Depression is widely known to negatively affect recovery after LLA and its association to suicidal tendencies is presumed, but highlighting this unique angle of the research could further strengthen the manuscript.

Title: Benefits, Acceptability, and Feasibility of Prosthetist Comorbidity Screening during Routine Care Visits: A Randomized Controlled Clinical Trial

- The title implies that CPOs are the primary participants in this study. However, the primary outcome is patient satisfaction. The title should be adjusted to more accurately reflect study population and outcome.

Abstract:

- Design may want to indicate that there was a mixed-methods approach incorporated as well since CPOs participated in focus groups.

Introduction:

- Citations 1 and 2 are very old and more current and accurate statistics in the amputation population are available. (e.g., Caruso M, Harrington S. Prevalence of limb loss and limb difference in the United States: implications for public policy. White Paper. Avalere and Amputation Coalition. 2024 Feb.; Meshkin DH, Zolper EG, Chang K, Bryant M, Bekeny JC, Evans KK, Attinger CE, Fan KL. Long-term mortality after nontraumatic major lower extremity amputation: a systematic review and meta-analysis. The Journal of Foot and Ankle Surgery. 2021 May 1;60(3):567-76.)
- L 73: How have EMRs negatively affected comorbidity screening? And is there any evidence, or lack of, regarding comorbidity screening in the LLA population? Expanding this information will bolster the rationale for the study.

Methods

- Why were bilateral amputees excluded?
- L113: The authors should include information regarding whether there was a minimal K level or ability to ambulate a minimal distance with/without assistance for study inclusion into the study.
- L119: It's unclear why it says, "received prosthetic componentry…in the last 3 months," but earlier states that participants had to be using a prosthesis for ≥ 1 year.
- L125 states "follow-up assessments." Were these items included in the initial assessment as well, making them inclusive of all assessments for SOC?
- L146: The authors should provide more rationale for why "suicidal ideation" is being screened. If there is any evidence or presumed connection to the high mortality rate is amputees, this should be elucidated in the Introduction of the manuscript.
- L156: Can the authors offer a citation/rationale for only testing 3 sites of the foot with monofilaments? Typically, at least 5 sites are recommended.
- L190: Was there a predetermined script for the focus groups? More detail on how the interviews were administered and qualitative data was analyzed should be included.
- Methods are somewhat confusing when looking at Fig. 1. Were patients seen at baseline then again at a 3-month follow up for all outcome measures? Or were all outcomes administered only at baseline and only surveys were followed up at 3 months? It's unclear how follow-up appointments were administered. Some were in person and some by phone? A clearer depiction of study procedure visits in Fig. 1 would be beneficial as "3-month observation via prosthetic medical chart" is confusing.

Results

- Is Table 1 representative of the initial visit only? The table should be labeled with time frame it presents.
- L285: A definition of what > 100% intervention fidelity is would be beneficial. Were certain methods more difficult to adhere to than others or was it random?

Discussion

- L387: Why would a visit that incorporates more outcomes (SOC+) be quicker at follow up appointments? The authors should expand upon this statement.

Figure 1 has a typo. It reads "heat attack."

VERSION 1 - AUTHOR RESPONSE

Reviewer: 1: Dr. David Landy, University of Kentucky

Comments to the Author:

1. Thanks for sharing this work. Seems to have been really well done and is a great step moving forward. Minor point but I view the value here not in that satisfaction increased with the intervention but rather that it did not decrease. I think the real value long-term here would be showing this could actually improve clinical outcomes. So the main value of this pilot is showing this intervention is feasible.

Thank you. We agree that it is important to highlight that satisfaction did not decrease, so we have modified the following statement in the 1st paragraph of the Discussion: *"Our primary hypothesis that SOC+ would result in greater patient satisfaction at three months and reduce prosthetic care minutes over the subsequent three months was not supported, <u>although notably satisfaction did not decrease with screening</u>."*

The following is included related to potential future value: *"Yet, future studies may consider alternative satisfaction surveys (<u>ideally with validation in adults post-amputation</u>) and extend follow-up windows beyond three-months to evaluate the impact of comorbidity screening on relevant health outcomes, e.g., limb wound development and subsequent amputation."*

2. A. The outcomes, satisfaction and time, fit with a pilot study in that they are continuous so easy to power, easy to measure, and don't require extended follow-up. With that said, it was not clear to me why these outcomes would necessarily be positive. I could imagine value in such a program and improving patient outcomes even if it did not save time or improve initial satisfaction. With that said, I do appreciate that satisfaction or rather lack of dissatisfaction and reasonable time add may be important to prostheses to get them to implement this.

Outcomes, i.e., patient satisfaction and reduced prosthetic care minutes in the 3 months post-baseline, were selected as these are important business metrics for prosthetic clinics that are easily collected as noted by the reviewer. Prosthetic

practices are not billing by visit like other healthcare providers, so unnecessary visits for issues that may be best addressed by other medical providers increases business expenses. We have added the following for clarity: "*Mitigating prosthetic visits for issues that may be best addressed by other medical providers reduces unnecessary business expenses as prosthetic practices receive bundled payments for the entire prosthetic process, i.e., practices are not billing by visit like other medical providers*."

Prosthetic practices with excellent patient satisfaction may be wary of practice change, so we agree that a lack of dissatisfaction with comorbidity screening is noteworthy. We also agree that long-term the greatest value of comorbidity screening by prosthetists to patients with amputations who are using prostheses may be related to other health outcomes, such as prevention of non-amputated "*limb wound development and subsequent amputation*" (as noted in the Study Strengths and Limitations section), but funding did not allow for a follow-up period adequate for evaluation of these downstream outcomes.

B. On that note, I'd mention that even though there was not a statistically significant difference in the times of the baseline visits (70 vs 76min), there likely is a difference. I think many clinicians will be surprised how long these visits are so adding 6min seems not clinically significant in this context. I think the modeling of predicting the times of other visits is complicated and may be straying a bit far.

We agree that a 6-minute difference is likely not clinically significance, which is highlighted by the statement: *"And, the addition of comorbidity screening did not significantly increase patient care time, suggesting it may be feasible during prosthetic follow-up visits."*

We respectfully request to keep the interaction analysis, as some may feel that a 6-minute difference is clinically relevant. But data helps to show "*that even though SOC+ is not initially quicker, it may help reduce time in subsequent follow-up appointments for those with longer baseline visits.*"

3. Please remove P values and hypothesis testing from Table 1 and the results text. While I appreciate you may have included these expecting a reviewer to ask for them, they are not ideal. If interested in discussion of this topic, please see https://pubmed.ncbi.nlm.nih.gov/35230980/ and https://pubmed.ncbi.nlm.nih.gov/37392076/.

This information has been removed as suggested.

Reviewer: 2: Prof. Natalie Vanicek, University of Hull
Comments to the Author:
bmjopen-2025-108623

1. This pilot RCT explores the feasibility and acceptability of comorbidity screening as part of routine care by prosthetists compared to usual care. The rationale is sound and would be of interest to professionals working within prosthetics therapies internationally. The authors cite that 87% of patients with

a LLA are cared for by prosthetists, but what about non-prosthesis users. They are unlikely to see prosthetists, and monitoring comorbidities in this group would be equally important, if not more important. Should there be standardised screening for comorbidities across all amputee patients? Please address this last point in the Discussion.

We have modified the Discussion to state the importance of comorbidity screening in all patients with amputation: *"Thus, hands-on clinical assessment of non-amputated limb health is critical in this patient population."*

The Study Strengths and Limitations section has also been modified: "*Results are from a single prosthetic practice and generalizable to adults with a unilateral transfemoral or transtibial amputation who receive prosthetic care; external generalizability may be enhanced with a multisite trial that includes a more heterogeneous LLA population and replication in practice settings that see both individuals who do and do not use a prosthesis."*

2. Introduction

Lines 63-64: when after amputation do depression and suicidal ideation exceed the general population? Does time since amputation affect these aspects of mental health?

We agree that time since amputation is likely a factor in mental health, as the individual may be grieving the loss of the limb and depression is one phase of the grief cycle. For clarity we have modified the sentence to highlight mental health issues persist beyond the acute post-amputation period: *"Additionally, more than a year after amputation, depression, suicidal ideation, and low back pain (LBP) rates exceed those of the general population, occurring in up to 30%,[1] 15%,[2] and 50%[3,4] of adults, respectively."*

3. Lines 72-73: why has the move to electronic medical records impacted comorbidity screening negatively?

This statement has been removed during the manuscript's modifications to allow space for clarification of other content given that the study previously cited was conducted in colorectal cancer screening and not related to the specific health screenings performed in our study.

4. Lines 87-88: I believe this statement is related to the Netherlands (reference 17) and this should be made clear as healthcare models vary greatly across countries.

This reference has been replaced with the following: "Pezzin LE, Dillingham TR, Mackenzie EJ, Ephraim P, Rossbach P. Use and satisfaction with prosthetic limb devices and related services. *Arch Phys Med Rehabil*. 2004;85(5):723-729. doi:10.1016/j.apmr.2003.06.002," which reported similar findings as Geertzen et al (2002), but surveyed individuals through the Amputee Coalition Registry, which is a United States-based organization.

5. Conversely, reference 18 relates to Iran so the authors should be cautious when making statements that may not be generalizable across countries' economic status vs healthcare models.

We agree that it would be ideal to have literature to cite from the United States (given our study is based in the United States), but, in general, literature related to prosthetics practice is minimal when compared to other healthcare disciplines. While we cite this work from Iran, this is also true in Sweden: (https://www.tandfonline.com/doi/full/10.1080/09638288.2024.2319342#abstract) and in the past, Amputee Coalition has reported that coordination of care is an area for improvement in post-amputation care based off survey data from the community at large. But, we have modified the statement to read: *"Patient satisfaction with prosthetist-orthotist care is generally high,[16] but one area for improvement may be care coordination with other providers.[17]"*

6. Methods
A. Line 116: why was prosthesis use of more than 1 year an inclusion criteria? Wouldn't monitoring comorbidities (e.g. depression) as early as possible be important for new amputees? Why were bilateral amputees excluded?

During the first year after amputation, individuals more frequently see their healthcare team members, as they have follow-up care related to the surgery, and there is a higher 'alert' level when it comes to identifying depression and 'sound' (non-amputated) limb issues. However, once individuals have stabilized in their definitive prosthesis, this attention wanes. To emphasize the focus on the post-acute period, the introduction was amended to state "*more than a year after amputation, depression, suicidal ideation and low back pain (LBP) rates exceed those of the general population*…"

Individuals with bilateral amputation were excluded due to the nature of the screening tests for 'sound' (non-amputated) limb health, i.e., monofilament testing and pedal pulse palpation necessitating presence of the contralateral limb. This has been clarified by the addition of the following statement: "*…contralateral LLA (due to lack of ability to assess non-amputated limb health),"*…

B. Lines 137-139: how was safety (i.e. falls prevention) ensured during the functional reach test?

For all performance-based outcome tests, participants were guarded by the prosthetist. This is now reflected by the addition of *"prosthetist guarding to ensure participant safety"* to the Methods.

C. Lines 141-142: did you monitor walking aid usage during the 10-metre walk test? If so, can you report it? If not, why not?

We have added specifics regarding assistive device use to Table 1. We have added the following to the text: "*…and use of an assistive device if needed (except for Functional Reach)."*

D. Line 163: how was participant anonymity (and/or protection of personal

identifiable details) ensured if findings were faxed to primary care centres? Fax seems a very outdated and insecure way of sharing sensitive data.

Many prosthetic practices, including the one involved in the clinical trial, still use fax, which with appropriate safeguards complies with privacy and security rules set by the Health Insurance Portability and Accountability Act (HIPPA). The letters faxed to the physician came from the prosthetic practice. There was no indication in the letter that the participant was involved in a research study. To clarify that this communication came from the practice and not the study team, the following statement was added: "*Screening results were documented in a templated letter on clinic letterhead, reviewed with the participant, and faxed to primary care by the prosthetist with the surveys attached.*'"

E. Line 170: has the client satisfaction inventory been validated for use in healthcare settings and/or with prosthesis users?

The Client Satisfaction Inventory has been validated for use in health services, more broadly. It has not been validated for use among adults with amputations. We have added the following to the text in the Study Strengths and Limitations section: "*…consider alternative satisfaction surveys (ideally with validation in adults post-amputation).*"

F. Lines 173-175: was patient-reported healthcare resource use cross-checked with the medical record data?

No, as the trial was conducted in a private prosthetic practice, researchers did not have access to medical record data beyond the prosthetic medical record. We have added to the Study Strengths and Limitations section: "*…and given lack of integrated healthcare records for our participants, self-reported healthcare utilization was unable to be verified with electronic medical records.*"

G. Line 183: what was the rationale for 17% attrition that was anticipated? What outcome measure was used to power the sample size calculation?

We have modified the statement to read: "*After accounting for 17% attrition given past research in LLA and prosthetic practice data,…*" We calculated the effect size from our target sample size (minus attrition), alpha (0.05) and power (0.80), with a goal of having a medium-to-large effect of the intervention (i.e., addition of comorbidity screening) on the primary outcomes.

H. Lines 188-189: did patients inform the choice of outcomes? Were they consulted in the design of the trial?

The prosthetists informed the choice of outcomes, but we have modified the Patient and Public Involvement Statement to include the following: "*Standardized baseline assessments were developed and agreed upon by the prosthetic team, which included one individual with an LLA.*"

I. Lines 191-192: why weren't focus groups done with patients as well as prosthetists to ascertain acceptability of the comorbidity screening?

The award that supported this trial was $25,000. We acknowledge that conducting focus groups with patient participants may have provided additional insight, but this was not feasible given funding available.

J. Can you describe the prosthetic site/practice that was used for this study. It's size, geographical catchment area, number (and type) of healthcare professionals working there, healthcare model, etc …..

The Study Sample section has been amended to include: "*at one of five Independence Prosthetics-Orthotics, Inc. offices in Delaware or Pennsylvania*,"… We have also added the following: *"The offices, which were independent from other medical facilities, were staffed by four certified prosthetists-orthotists (1 male; 3 females), who also consented to research participation."*

7. Results
A. Line 219: it seems strange that the sample had a higher functional mobility level when the majority of amputees tend to be older and have had their amputation related to disease and therefore have lower mobility levels.

This is likely a result of the sampling methodology. While we offered all individuals presenting to clinic with unilateral transfemoral or transtibial amputation the opportunity to participate in the study to mitigate sample selection bias, individuals with lower functional mobility may frequent prosthetic clinics less and/or not use a prosthesis and thus, not seek out routine prosthetic services. Further, mobility limitations and transportation insecurity are known healthcare access barriers in this patient population that likely disproportionately affect those who are older, with greater medical comorbidities, and with lower functional mobility levels. We have modified the Study Strengths and Limitations section: *"Results are from a single prosthetic practice and generalizable to adults with a unilateral transfemoral or transtibial amputation who receive prosthetic care; external generalizability may be enhanced with a multisite trial that includes a more heterogeneous LLA population (e.g., lower functional mobility levels) and replication in practice settings that see both individuals who do and do not use a prosthesis."*

B. Throughout this section, please spell out numbers when twelve or less. E.g. Three SOC+ individuals …..

This has been corrected throughout the paper for consistency.

C. Lines 278-279 and lines 306-308: prosthetists reported feeling uncomfortable dealing with patient-reported feelings of depression and/or suicide. Did the prosthetics centres have "in-house" counsellors and/or psychologists? This could make the prosthetists more comfortable screening for these mental health issues if experts were "at hand" to help patients deal with them.

Similar to private outpatient physical therapy practices, private prosthetic-orthotic practices typically do not have in-house mental health services and must refer to another medical practice. We have clarified that offices were "*independent from other medical facilities*," e.g., not embedded within a hospital system.

D. Line 289-290: why was shortness of breath (in itself) an adverse event? Did it lead to further complications?

Adverse events were defined as "*any unfavorable or untoward medical occurrence in a participant, including any abnormal sign, symptom, or disease associated with research involvement, whether or not the event was considered related to research participation.*"

Shortness of breath, an unfavorable medical symptom, was experienced during the Timed Up and Go and necessitating holding the 10-Meter Walk Test, although the shortness of breath resolved within the session. We have clarified that this event "*resolved during the visit*."

8. Discussion
A. Lines 315-326: this seems like reporting of results. Could the numerical results be summarised in one of the tables (and then referred to here) so that the narrative is more critically reflective in the Discussion section.

As suggested, data are now summarized in Table 1 and the Discussion has been revised, using the data to demonstrate the importance of hands-on clinical assessment.

B. According to the functional performance-based outcome data, your sample was high functioning. Did you not collect data regarding K-levels? Can you report it? Can you comment further on the implications of sampling a high functioning group of patients vs. mostly K1-K2 users? You touch on this lightly in Line 219, but it needs addressing in the Discussion too.

K-level has been added to Table 1. We have modified the following statement in the Discussion's Study Strengths and Limitations section: "*…external generalizability may be enhanced with a multisite trial that includes a more heterogeneous LLA population (e.g., lower functional mobility levels)…*"

9. Figure 1 should be titled as a CONSORT flowchart

In reviewing CONSORT templates, we believe that "Figure 1. Study Flow Diagram" includes information reported beyond CONSORT recommendations (e.g., training, fidelity), therefore, we would prefer not to include CONSORT in the title of this Figure.

Reviewer: 3: Dr. Sheila M. Clemens, Florida International University

Comments to the Author:

1A. This study addresses an important area of clinical care that is understudied in the LLA population and offers the foundation for future research to improve care of patients. Tracking comorbidities over time is an important, understudied area of LLA research and this study offers information to fill gaps in knowledge. Additionally, this study provide a basis for future clinically implementable assessments to potentially enhance care of patients with LLA.

Thank you for acknowledging the contribution of this work to existing literature.

1B. However, the Methods of the study to include baseline and follow up visits are unclear and would benefit from significant revision to improve reader understanding.

Methods in the manuscript have been revised to better delineate between baseline assessment visits and remote follow-up.

1C. Some of the most interesting findings resulted from the CPO interviews and realistic clinical implications for the screenings.

We agree. As such, we have a section dedicated to the 'Prosthetist Perspective' and the Discussion suggests areas for improvement for future work, which may be necessary to evoke practice change in prosthetic clinics.

1D. Furthermore, the manuscript frequently alludes to suicide an important comorbidity, and this topic is understudied in amputation research. However, the study rationale doesn't reflect on why it's important to screen for suicide risk in the LLA population in particular, or the urgency by which it should be addressed. Depression is widely known to negatively affect recovery after LLA and its association to suicidal tendencies is presumed, but highlighting this unique angle of the research could further strengthen the manuscript.

We have added this to the Discussion: *"Prosthetist training in depression screening pairs well with suicidal ideation screening since suicidal ideation may occur concurrently with depressive symptoms* *<u>but receives less attention (and is included on the PHQ-9)</u>."*

2. Title: Benefits, Acceptability, and Feasibility of Prosthetist Comorbidity Screening during Routine Care Visits: A Randomized Controlled Clinical Trial
• The title implies that CPOs are the primary participants in this study. However, the primary outcome is patient satisfaction. The title should be adjusted to more accurately reflect study population and outcome.

Prosthetists were participants in the study and satisfaction, is in part, dependent on prosthetist interactions with the patient, but we agree that prosthetists were not the only participants. The manuscript has been retitled: *<u>"Prosthetist Screening for Comorbidity during Routine Care Visits: A Randomized Controlled Trial Evaluating Benefits, Acceptability, and Feasibility</u>".*

3. Abstract:
• Design may want to indicate that there was a mixed-methods approach incorporated as well since CPOs participated in focus groups.

Added "*<u>with mixed-methods approach</u>*" to Abstract.

4. Introduction:
A. Citations 1 and 2 are very old and more current and accurate statistics in

the amputation population are available. (e.g., Caruso M, Harrington S. Prevalence of limb loss and limb difference in the United States: implications for public policy. White Paper. Avalere and Amputation Coalition. 2024 Feb.; Meshkin DH, Zolper EG, Chang K, Bryant M, Bekeny JC, Evans KK, Attinger CE, Fan KL. Long-term mortality after nontraumatic major lower extremity amputation: a systematic review and meta-analysis. The Journal of Foot and Ankle Surgery. 2021 May 1;60(3):567-76.)

The Caruso and Harrington reference provides prevalence, not incidence data; we desired to share incidence data (inception timepoint) as the subsequent part of sentence talks about mortality with respect to lower-limb amputation incidence. We have opted not to use the Meshkin, et al. reference as this is specific to non-traumatic lower-limb amputation and not inclusive of all-cause lower-limb amputation, which is the demographic for our study. We are open to more contemporary references that convey all-cause lower-limb amputation incidence and subsequent mortality but did not find any during a subsequent literature search, therefore, we have retained the original references.

B. L 73: How have EMRs negatively affected comorbidity screening? And is there any evidence, or lack of, regarding comorbidity screening in the LLA population? Expanding this information will bolster the rationale for the study.

We have removed the statement/reference related to electronic medical record (EMRs); see response to Reviewer 2, Item 3 above. During our literature review, we found no evidence of hands-on comorbidity screening in clinical practice among adults with lower-limb amputation (LLA). Most studies exploring comorbidity are longitudinal cohort studies. To highlight this finding, we have modified the first sentence of the Discussion to: "*Most studies on comorbidity in LLA are longitudinal cohort studies. This is the first randomized, controlled clinical trial evaluating comorbidity screening potential benefits, acceptability, and feasibility during routine prosthetic care visits.*"

4. Methods
A. Why were bilateral amputees excluded?

As noted above, individuals with bilateral amputation were excluded due to the nature of the screening tests for 'sound' limb health necessitating presence of the contralateral limb – i.e., monofilament testing, pedal pulse palpation. This has been clarified by the addition of the following statement: "*…contralateral LLA (due to lack of ability to assess non-amputated limb health),"…*

B. L113: The authors should include information regarding whether there was a minimal K level or ability to ambulate a minimal distance with/without assistance for study inclusion into the study.

We have clarified in the manuscript that there was no criteria related to functional mobility (i.e., K-level)/ambulatory status: "*…who reported using their prosthesis for ≥ one–year (regardless of ambulatory status).*"

C. L119: It's unclear why it says, "received prosthetic componentry...in the last

3 months," but earlier states that participants had to be using a prosthesis for ≥ 1 year.

Individuals who had received prosthetic componentry in the preceding 3 months would be presenting for prosthetic adjustments, not for routine follow-up care. Additionally, receipt of a new prosthetic component/device in the preceding 3 months would directly influence 3-month care utilization prior to the baseline visit. For these reasons, those individuals were excluded. For clarity, the statement has been modified to: *…"received new or replacement prosthetic componentry in the last three months due to the potential confounding influence on three-month prosthetic care utilization."*

D. L125 states "follow-up assessments." Were these items included in the initial assessment as well, making them inclusive of all assessments for SOC?

Individuals enrolling in the study were presenting for routine follow-up care – the "*standardized follow-up assessment*" is meant to indicate that initial baseline visit – the sentence has been revised to state "*standardized routine care assessments*" to better differentiate between subsequent prosthetic follow-up in-person care and 3-month remote research follow-up.

E. L146: The authors should provide more rationale for why "suicidal ideation" is being screened. If there is any evidence or presumed connection to the high mortality rate is amputees, this should be elucidated in the Introduction of the manuscript.

The Patient Health Questionnaire-9 item is widely used in primary care to screen for major depression in the United States due to its psychometric properties; the 9th item on this questionnaire asks about suicidal ideation. If a patient endorses this 9th item, it requires immediate action, so by default providers must pay attention to not just the total score (which reflects depressive symptoms) but the response to this 9th item (i.e., suicidal ideation) and act when indicated. We have added the following to the Discussion: "*Prosthetist training in depression screening pairs well with suicidal ideation screening since suicidal ideation may occur concurrently with depressive symptoms but receives less attention (and is included on the PHQ-9).*"

To our knowledge, there is no recent, existing literature (last 40 years) linking suicide to the higher mortality rates seen in the United States among patients who are post-acute lower-limb amputation.

F. L156: Can the authors offer a citation/rationale for only testing 3 sites of the foot with monofilaments? Typically, at least 5 sites are recommended.

According to a systematic review conducted by Feng et al (doi: 10.1016/j.jvs.2009.05.017), a three-site test of the great toe, 3rd metatarsal, and 5th metatarsal maximizes the diagnostic value of monofilament testing. This citation has been added to the sentence describing sites tested: "*Protective sensation was evaluated using a 5.07/10g monofilament applied to the plantar aspect of the great toe and the third and fifth metatarsal heads, while avoiding calluses for up to three trials per site.*[29]"

G. L190: Was there a predetermined script for the focus groups? More detail on how the interviews were administered and qualitative data was analyzed should be included.

We have added the following file to the manuscript: *"Supplemental Figure 3*: Prosthetist Focus Group Script", which provides details regarding how the interview was administered and how data was handled to supplement the text previously provided: *"To evaluate prosthetist acceptability of comorbidity screening, using a semi-structured interview format, the principal investigator conducted a two-hour focus group in December of 2022. Data was recorded by two research team members, summarized by the principal investigator, and reviewed for accuracy by two prosthetists."*

5. Methods are somewhat confusing when looking at Fig. 1. Were patients seen at baseline then again at a 3-month follow up for all outcome measures? Or were all outcomes administered only at baseline and only surveys were followed up at 3 months? It's unclear how follow-up appointments were administered. Some were in person and some by phone? A clearer depiction of study procedure visits in Fig. 1 would be beneficial as "3-month observation via prosthetic medical chart" is confusing.

Figure 1 has been modified to delineate onsite versus remote components and the text has been modified to *"Extracted Onsite Prosthetic Electronic Medical Record Data from Assessment Visit to Remote Follow-Up (i.e., 3 months)".* There was no in-person visit at the 3-month follow-up; this was strictly remote. Manuscript text has been modified to assist with clarification of onsite versus remote components. For example, the Methods now read: *"Via RedCap, all participants were requested to complete the PHQ-9 and the STarT remotely at three-month follow-up (see Figure 1). Participants also completed two satisfaction surveys."*

6. Results
A. Is Table 1 representative of the initial visit only? The table should be labeled with time frame it presents.

Table title has been revised to "*Participant Characteristics at Baseline Assessment Visit*".

B. L285: A definition of what > 100% intervention fidelity is would be beneficial. Were certain methods more difficult to adhere to than others or was it random?

Supplemental Files 1 (for chart reviews) and 2 (for onsite assessments) provide criteria used to determine fidelity; scoring for fidelity is available at the bottom of these files. Scores of >100% are not possible. While certain items/procedures/processes appear to be more challenging to adhere to than others in clinical practice (i.e., items conducted outside of patient care time versus within patient care time), given only 4 prosthetists and overall high fidelity, we have refrained from individual item analyses.

7. Discussion

A. L387: Why would a visit that incorporates more outcomes (SOC+) be quicker at follow up appointments? The authors should expand upon this statement.

Added the following example: *"For example, an individual referred for >low-risk LBP may no longer misattribute the LBP to prosthetic malalignment alone."*

8. Figure 1 has a typo. It reads "heat attack."
This has been corrected to *"heart attack".*

VERSION 2 - REVIEW

Reviewer **1**

Name **Landy, David**

Affiliation **University of Kentucky, Department of Orthopaedic Surgery**

Date **18-Jan-2026**

COI

Thank you for addressing the reviewer comments.

Reviewer **2**

Name **Vanicek, Natalie**

Affiliation **University of Hull, Sport, Health and Exercise Science**

Date **06-Jan-2026**

COI

The authors have addressed all of my comments and I do not have further concerns. Thank you.

Reviewer **3**

Name **Clemens, Sheila M.**

Affiliation **Florida International University**

Date **18-Jan-2026**

COI

Thank you for the opportunity to review this revised manuscript. Overall, the authors have satisfactorily addressed concerns and the manuscript is improved. I have a couple of minor

suggestions in areas that remain problematic but overall think this is important and insightful work and look forward to its eventual publication.

Results

L229: Though I agree with Reviewer 1's recommendation to not include p values (since there were no significant differences), I feel that the statement "There were no significant between group differences" should be maintained in the manuscript to clarify the data for the reader. Otherwise, I think the reader would logically question whether or not differences existed between the groups on the variables in Table 1.

L409: The addition of the sentence, "For example, an individual referred for >low-risk LBP may no longer misattribute the LBP to prosthetic malalignment alone," is confusing. To whom is the patient being referred to for LBP? The prosthetist or a rehab specialist? The authors may want to revise this sentence to better communicate to the reader why a follow up appointment in the SOC+ group would be quicker.

VERSION 2 - AUTHOR RESPONSE

Reviewer: 3
Dr. Sheila M. Clemens, Florida International University
Comments to the Author:
Thank you for the opportunity to review this revised manuscript. Overall, the authors have satisfactorily addressed concerns and the manuscript is improved. I have a couple of minor suggestions in areas that remain problematic but overall think this is important and insightful work and look forward to its eventual publication.

Results
1A. L229: Though I agree with Reviewer 1's recommendation to not include p values (since there were no significant differences), I feel that the statement "There were no significant between group differences" should be maintained in the manuscript to clarify the data for the reader. Otherwise, I think the reader would logically question whether or not differences existed between the groups on the variables in Table 1.

We have readded the following statement to the Results: "There were no significant between-group differences in participant demographics or data obtained from standardized clinical evaluations (Table 1; p>.050)."

1B. L409: The addition of the sentence, "For example, an individual referred for >low-risk LBP may no longer misattribute the LBP to prosthetic malalignment alone," is confusing. To whom is the patient being referred to for LBP? The prosthetist or a rehab specialist? The authors may want to revise this sentence to better communicate to the reader why a follow up appointment in the SOC+ group would be quicker.

We have modified this section to the following:

“For example, an individual classified as >low-risk LBP may require treatment addressing both biological and psychological aspects of their pain experience and may be referred for comprehensive physical therapy that addresses thoughts and behaviors related to LBP in addition to biological factors. During subsequent prosthetic visits, patients may be less likely to erroneously contribute their LBP to biological factors, like prosthetic alignment, when psychological factors may be greater contributors to their LBP experience.”
